# Evaluation of recruitment methods for a trial targeting childhood obesity: Families for Health randomised controlled trial

**DOI:** 10.1186/s13063-015-1062-x

**Published:** 2015-11-25

**Authors:** J. Fleming, A. Kamal, E. Harrison, T. Hamborg, S. Stewart-Brown, M. Thorogood, F. Griffiths, W. Robertson

**Affiliations:** Warwick Medical School, University of Warwick, Coventry, CV4 7AL UK; Division of Health Sciences, Warwick Medical School, University of Warwick, Coventry, CV4 7AL UK

**Keywords:** Childhood obesity, weight management, randomised controlled trial, recruitment

## Abstract

**Background:**

Recruitment to trials evaluating the effectiveness of childhood obesity management interventions is challenging. We report our experience of recruitment to the Families for Health study, a randomised controlled trial evaluating the effectiveness of a family-based community programme for children aged 6–11 years, versus usual care. We evaluated the effectiveness of active recruitment (contacting eligible families directly) versus passive recruitment (informing the community through flyers, public events, media).

**Methods:**

Initial approaches included passive recruitment via the media (newspapers and radio) and two active recruitment methods: National Child Measurement Programme (letters to families with overweight children) and referrals from health-care professionals. With slow initial recruitment, further strategies were employed, including active (e.g. targeted letters from general practices) and passive (e.g. flyers, posters and public events) methods. At first enquiry from a potential participant, families were asked where they heard about the study. Further quantitative (questionnaire) and qualitative data (one-to-one interviews with parents/carers), were collected from recruited families at baseline and 3-month follow-up and included questions about recruitment.

**Results:**

In total, 194 families enquired about Families for Health, and 115 (59.3 %) were recruited and randomised. Active recruitment yielded 85 enquiries, with 43 families recruited (50.6 %); passive recruitment yielded 99 enquiries with 72 families recruited (72.7 %). Information seen at schools or GP surgeries accounted for over a quarter of enquiries (28.4 %) and over a third (37.4 %) of final recruitment. Eight out of ten families who enquired this way were recruited. Media-led enquiries were low (5 %), but all were recruited. Children of families recruited actively were more likely to be Asian or mixed race. Despite extensive recruitment methods, the trial did not recruit as planned, and was awarded a no-cost extension to complete the 12-month follow-up.

**Conclusions:**

The higher number of participants recruited through passive methods may be due to the large number of potential participants these methods reached and because participants may see the information more than once. Recruiting to a child obesity treatment study is complex and it is advisable to use multiple recruitment strategies, some aiming at blanket coverage and some targeted at families with children who are overweight.

**Trial registration:**

Current Controlled Trials ISRCTN45032201 (Date: 18 August 2011)

**Electronic supplementary material:**

The online version of this article (doi:10.1186/s13063-015-1062-x) contains supplementary material, which is available to authorized users.

## Background

Recruiting participants is one of the most challenging parts of carrying out trials. To take part, participants must meet eligibility criteria, be willing to be randomised into a treatment or comparison group, adhere to the study conditions, and participate in the required data collection [1]. It is important that sufficient numbers of participants are recruited to studies and that attrition rates remain low [2]. Poor recruitment reduces the power of a trial, which may make the results inconclusive. Biased recruitment will mean that the participants are unrepresentative of the population, while slow recruitment affects delivery of the intervention, especially in a trial using a group-based intervention. Problems with recruitment may increase the cost of a trial. Recruiting participants to health research studies is resource intensive and is recognised as a challenge [3]. In a review of 114 trials funded by two UK funding agencies, less than a third of the trials achieved their original recruitment target and half were awarded a time extension [4]. The start of recruitment was delayed in 41 % of trials and early recruitment problems were identified in 63 %. The interrelationship between trial features and recruitment success was complex [4]. Researchers may adopt different strategies of varying costs to reach their participant target.

Our trial aimed to recruit families with obese children to a group intervention. In recent years, the prevention and management of childhood obesity have become a public health priority, and a growing number of trials are testing interventions for prevention [5] and treatment [6]. The most recent Health Survey for England found that 13 % of boys and 12 % of girls in England aged 2–10 were obese in 2013, and a further 14 % and 13 %, respectively, were classified as overweight [7].

A number of other studies on childhood obesity have studied the success of their recruitment methods. Recruitment methods can be categorised as active, in which researchers target potentially eligible participants, such as with a targeted letter or referral from a health professional, or as passive, in which researchers inform the whole community using flyers, posters, public events and media. A study from the USA evaluating active versus passive recruitment for parent–child pairs in two child obesity intervention trials [8] showed that active recruitment methods, such as paediatric referral and targeted mailings, led to a higher number of recruited participants but required significant resources. The Loozit randomised controlled trial also reported the effectiveness of strategies to recruit overweight and obese adolescents aged 13–16 years from the community to a weight management treatment trial [9]. Out of 474 enquiries, 32 % resulted in an enrolment to the trial. Passive methods via local newspapers and school newsletters accounted for nearly 60 % of enquiries and enrolments, and were the most cost-effective recruitment strategies [9]. The Families for Health pilot study found that self-referral following articles in the local media led to a higher number of recruited participants and higher completion rates than recruitment via health professionals [10].

In a recent physical activity trial, researchers found that recruiting opportunistically (approaching patients in the waiting room who were attending routine general practice (GP) appointments) more than halved the mean participant recruitment time compared to a systematic recruitment approach (GPs selecting eligible patients from practice lists), but participants recruited this way were four times more likely to withdraw from the study or be lost to follow-up [11]. The authors indicated that this higher dropout rate could be due to confounding with those waiting for a GP appointment being in poorer general health, or be due to having less time to consider whether they really wanted to take part leading to greater dropout.

Research to date demonstrates the importance of using appropriate recruitment methods to attain the required number of participants into a trial. This paper aims to compare the outcome of different recruitment strategies in a family-based childhood obesity treatment trial, using data from the Families for Health trial, and make recommendations for recruitment in similar future trials.

## Methods

First we describe the Families for Health trial then focus on the methods we used to recruit participants and evaluate the different recruitment methods.

### The families for health study design

Families for Health is a family-based group intervention for the treatment of children aged 6–11 who are overweight or obese. The intervention is 10 weeks long, 2½ hours per week, run in a community venue with parents/carers and children attending parallel groups. Four trained facilitators, two for the children’s group and two for the parents/carers group, run each programme. In contrast to other similar interventions, the Families for Health programme emphasises parenting skills, relationship skills and emotional and social development, combined with information about lifestyle. The Families for Health trial evaluated the effectiveness and cost-effectiveness at 12 months of the Families for Health programme delivered in the NHS using a randomised controlled trial. Participants across three former West Midlands NHS Primary Care Trusts (Sites A, B and C) were randomly allocated to receive the Families for Health programme or usual care. The aim was to run six Families for Health programmes (two in each site). Usual care could vary across the three sites, but consisted of either a community-based group intervention or a one-to-one intervention. Ethical approval for the study was obtained from the National Research Ethics Services (NRES) Committee West Midlands – Coventry & Warwickshire Research Ethics Committee (REC), (reference 11/WM/0290). Further study and intervention details are published elsewhere [12].

#### Inclusion criteria

The trial aimed to recruit 120 families (40 from each site) over 12 months (between March 2012 to February 2013) [12]. The initial inclusion criteria stipulated that families must have at least one overweight (≥91st centile for BMI) or obese (≥98th centile for BMI) child aged 7–11 years, based on the UK 1990 BMI charts [13]. Families were excluded where the parent/carer or child had insufficient command of English to be able to participate in the group, if the child had a metabolic or other recognised medical cause of obesity, or if the child had severe learning difficulties and/or behavioural problems that would make participation difficult.

Researchers used a three-step procedure to obtain informed consent, giving parents/carers and children time to consider whether they wished to participate [12]. First, after each enquiry, potential participants were given or sent by post information sheets about the trial (child and parent versions). Second, after a minimum of 3 days, parents/carers were contacted by telephone to ask whether they were interested in taking part in the trial and to answer any questions. Third, a researcher visited the parent/carer(s) and child(ren) at their home and obtained the parents/carers’ written consent and the child’s written assent. Researchers were trained in informed consent. Informed consent was obtained from all randomised participants.

#### Planned recruitment strategies

Recruitment of families started in mid-March 2012 across Sites A and B, while recruitment in Site C was delayed until mid-April 2012 due to delays in setting up the intervention. We used passive recruitment using the media (advertisements in local newspapers and on local radio) and active targeted recruitment both by sending letters to families with an overweight or very overweight child recently measured in the National Child Measurement Programme (NCMP) and by referrals of relevant families from health-care professionals.

#### Changes to eligibility criteria

In the first month of recruitment, some parents of 6-year-old children were keen to take part, and were disappointed when told they were not eligible. The programme suits younger children, with its focus on active games, the activities around healthy eating and circle time. We decided to change the inclusion criteria to children aged 6–11 years, to allow 6-year-olds to take part. This change was approved by the funders, National Institute for Health Research (NIHR) Health Technology Assessment (HTA), and received ethical approval.

#### Changes to projected recruitment rate

**Fig. 1:**
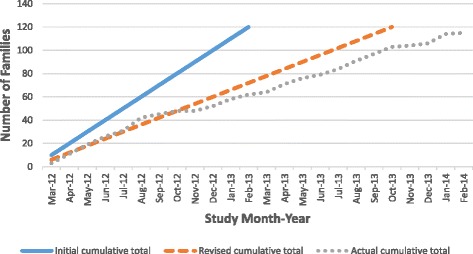
Participant recruitment to Families for Health study

Recruitment was intended to be completed in 12 months, but instead took 24 months. In April 2013, because of slow recruitment, the target recruitment rate was changed from ten to six families per month. Recruitment in Site A was extended to February 2014, running an additional seventh Families for Health programme to increase the number of study participants. We finally recruited 115 families, five short of the original target. Figure 1 shows the projected graph of planned recruitment at the start of the study with a target of recruiting ten families per month, the revised plan for recruitment of six families per month, and the actual cumulative total.

#### Changes to recruitment methods

Because of the slow recruitment rate, we adopted additional methods of recruitment. These included, as active methods, targeted letters from GPs to families identified in their records as having an overweight or obese child in Site C and similar letters from local Change4Life advisors in Site B. (Change4Life is a campaign in England and Wales with information aimed at families to encourage healthy eating and physical activity: www.nhs.uk/Change4Life/). Additional passive methods included placing flyers and posters at schools and in school newsletters, in the community and at GP surgeries and the research team attending public events to raise awareness of the trial. As recruitment continued, we found that word of mouth became a useful recruitment method. Additional file 1 gives examples of the recruitment methods.

### Design of recruitment study

#### Data collection

At the first enquiry from a potential participating family, the parent/carer was asked where they heard about the study. For health professional referrals, the source of the referral was noted. Once a family was recruited, we collected quantitative and qualitative data at baseline, and the 3-month and 12-month follow-ups. At the baseline visits, participants completed a recruitment questionnaire, which included the question, ‘Where did you hear about the research study?’ (GP, school nurse, health visitor, paediatrician, school, media, flyer/leaflet, friends/family, letter from NCMP, other). During analysis, in cases where there was a discrepancy between the questionnaire response and the response given when they first enquired about the study, the response at their first enquiry was used. Where parents/carers cited more than one source of information about the study, we recorded the method that was the final prompt for them to enquire about the study with the research team.

One-to-one semi-structured interviews were undertaken with parents/carers during a control family’s 3-month follow-up visit or an intervention family’s interview after accessing a Families for Health programme (at 3 months from baseline, or later if there was a delay in accessing the intervention). Consecutive sampling included representation of all Families for Health groups aiming for diversity of age, ethnicity and gender of the children, family size and whether they completed the intervention or not. The mean duration of parent/carer interviews was 20 minutes (range 4 to 62 minutes). Interviews were digitally recorded, subject to the permission of each participant, and transcribed verbatim.

#### Data analysis

Transcripts were checked for accuracy against the initial recording. All transcripts were anonymised, and data were analysed using NVivo 10. Coding of data was thematic, based on the interview schedules with the addition of emergent themes [14]. We developed a coding framework during meetings of the research team, ensuring that important topics in the data were captured. The initial codes were then developed into themes and reviewed again in comparison with the coded extracts [15]. At least 10 % of the coded data was cross-checked by another member of the research team, to reduce researcher bias. For this paper, we report those data coded under ‘Why joined the study/intervention’, which included anything to do with how the family had heard about the study and what motivated them to take part.

Family attendance for the Families for Health intervention was recorded each week by the facilitators. At follow-up visits, participants were asked what intervention they attended and how many sessions. For the purposes of this paper, the attendance records completed by facilitators are used for Families for Health participants, and self-reported attendance for families in the usual care arm.

Statistical analysis involved comparing the baseline characteristics of those recruited by active and passive methods. A chi-squared test was used to compare categorical variable baseline characteristics, unless 20 % of the expected frequencies of categories was less than 6, in which case Fisher’s exact test was used. Differences in continuous variable baseline characteristics between actively and passively recruited participants were compared by means of independent sample T-tests.

## Results

During the study we broadened the range of recruitment methods we used in response to low recruitment rates as described. We now present data on the success of recruitment strategies, comparing passive and active methods, along with qualitative data that provide insights into how the different methods of recruitment work.

### Comparison of recruitment methods

A total of 194 families enquired about the study and were sent further information by post or email. Information on where the family heard about the study was available for 184 families. Overall, 115 families went on to be recruited and randomised. Table 1 presents recruitment methods and the outcomes of each method. Active recruitment yielded 85 potential participants, with 43 recruited (50.6 %); passive recruitment yielded 99 potential participants, with 72 recruited (72.7 %). Passive recruitment not only resulted in a higher proportion of enquires being recruited to the study (passive: 72.7 % vs active: 50.6 %, *p* < 0.002), but also a higher proportion of the total number of families recruited (passive: 72/115 vs active: 43/115, *p* < 0.007).

**Table 1:** Recruitment methods and outcomes

Recruitment method	Amount or number of times	Associated cost of recruitment method	Number of families enquiring about the study^a^	Number of families recruited	Enquiries who were recruited
Active methods					
Referral from health professional	Health professionals (e.g. school nurses, hospital and community paediatricians, GPs, dieticians) were sent information and some professional meetings were attended by researchers (all sites)	Cost to health professional: excess treatment costs	69	30	43.5 %
	65 health professionals telephoned us with details of a potential family (from across all sites)				
	On four occasions, a researcher attended an obesity clinic and the doctor referred families to the researcher (hospital covering Sites A and B)	Researcher time: 4 × ½ day plus travel			
Targeted letter from health professional	Families for Health information provided with NCMP letter to overweight and obese children from year 6 (age 10–11 years) (*n* = approximately 600, from both Site A and Site C)	Postage	16	13	81.3 %
	50 letters were sent by GPs to families of children identified on GP lists as being overweight or obese (Site C)				
	50 letters sent by Change4Life advisor to families on their case list (Site B)				
Sub-total			85	43	50.6 %
Passive methods					
School (poster, newsletter, flyer)	All primary schools in Sites B and C contacted twice, in Site C three times.	Researcher time for first contact: 2 days, second and third: ½ day	30	24	80 %
	Contacts were a phone call on the first occasion, then emails. Flyers were sent out as requested by the schools. All schools were sent text that could be included in a school newsletter.				
		Postage or travel for delivering flyers			
GP/hospital (poster, flyer)	Posters and flyers were sent to local primary-care surgeries and hospitals × 2 (all sites)	Postage	25	19	76 %
Community (poster, flyer)	Posters were sent to community venues such as libraries, children’s centres and leisure centres × 3 (all sites)	Postage	5	4	80 %
Flyer (unspecified)	Flyers distributed using methods described above but no details from potential participants where flyer was seen		6	2	33 %
Public events	9 events (10 days) across all sites: Site A: 2 events; Site B: 2 events: Site C: 5 events (6 days)	Researcher time (10 days) plus travel to event	17	9	52.9 %
	Researcher attended public health and community events. This involved displaying information about the study, and scales and height meter to measure potential participants’ BMI. Families who had children with a high BMI were given further details of the study and contact details were taken if the family was interested in taking part.				
Media (newspaper, radio, internet)	4 radio interviews (Sites A and B)	Free	9	9	100 %
	2 paid newspaper articles (Site C)	Cost of articles			
	5 free newspaper or magazine articles (all sites)	No cost incurred			
	Families for Health website	No cost incurred			
	NHS and local authority website, Twitter (all sites)	No cost incurred			
	Local newspaper Twitter feed	No cost incurred			
	3 adverts in Primary Care Research Network (PCRN) newsletter	No cost incurred			
Word of mouth	–	No cost incurred	7	5	71.4 %
Sub-total			99	72	72.7 %
Total			184	115	62.5 %

^a^Ten unknown (not included in figures)

The most productive active method of recruitment was a referral from a health professional, where 69 enquiries about the study resulted in 30 families recruited to the study. Information seen at school or GP surgery accounted for 55 enquiries and over a third (37.4 %) of final recruitment. Media-generated enquiries were few, but all nine were recruited.

The 79 participants not recruited to the study comprised 42 who had been identified through active methods, 27 identified through passive methods and 10 where the source of the enquiry was not known. The most common reason for them not being recruited was that we were unable to contact them after their initial enquiry about the study (18, 22.8 %), followed by not being eligible to take part in the study (14, 17.7 %). Some of the families (10, 12.7 %) were excluded because they were unable to attend on a Saturday when the intervention groups were to be run. Reasons given by potential participants for non-recruitment included: ‘could not commit to a full 10-week programme’, ‘too far to commute to intervention, including cost implications’ and ‘family has more important issues going on and do not feel it is the right time to tackle child’s weight, such as school issues, family bereavement’.

Table 2 shows the baseline characteristics of families recruited by the active and passive methods. The children recruited through active recruitment methods were slightly older and had a higher baseline mean BMI, but there was no difference in BMI z-score. The baseline BMI of parents/carers of children recruited through passive methods was significantly higher (*p* = 0.026). A higher proportion of Asian and mixed-race children were recruited through active methods. There was no significant difference in programme attendance or completion.

**Table 2:** Baseline characteristics of participants recruited using active vs passive methods

Baseline characteristic	Active recruitment	Passive recruitment	*p* value of difference
Number of families	43 (37.4 %)	72 (62.6 %)	0.007
Number of parents/carers	52 (38.0 %)	85 (62.0 %)	0.005
Number of children	46 (35.9 %)	82 (64.1 %)	0.002
Gender of children			0.616
Boys	24 (52.2 %)	39 (47.6 %)	
Girls	22 (47.8 %)	43 (52.4 %)	
Mean age of child (years) (SD)	9.91 (1.61)	9.18 (1.52)	0.012
Mean age of parent/carer (years) (SD)	39.59 (7.19)	40.46 (7.86)	0.517
Family type^a^			0.162
Two-parent family	20 (46.5 %)	40 (55.6 %)	
Single parent (mother)	22 (51.2 %)	24 (33.3 %)	
Single parent (father)	0 (0 %)	0 (0 %)	
Step-family	1 (2.3 %)	6 (8.3 %)	
Other (e.g. living with other relative)	0 (0 %)	2 (2.8 %)	
Child ethnicity^a^			0.002
White	24 (52.2 %)	55 (67.1 %)	
Black	0 (0 %)	10 (12.2 %)	
Asian	13 (28.3 %)	9 (11.0 %)	
Chinese	0 (0 %)	0 (0 %)	
Mixed	9 (19.5 %)	7 (8.5 %)	
Other	0 (0 %)	1 (1.2 %)	
Baseline mean (SD) BMI of child	27.02 (4.56)	25.21 (4.13)	0.024
Baseline BMI z-score (SD) of child	2.80 (0.85)	2.66 (0.81)	0.273
Baseline mean (SD) BMI of parent/carer	30.19 (5.93)	33.01 (8.51)	0.026
Socio-economic status *n* (%)			0.361
Class 1 (higher managerial, administrative and professional occupations)	14 (32.5 %)	25 (34.7 %)	
Class 2 (intermediate occupations)	9 (20.9 %)	10 (13.9 %)	
Class 3 (routine and manual occupations)	10 (23.3 %)	26 (36.1 %)	
Class 4 (never worked)	10 (23.3 %)	11 (15.3 %)	
Families for Health Attendance (*n* = 56)			
DNA^a^	3 (16.7 %)	11 (29.0 %)	0.169
Attended at least one session^a^	15 (83.3 %)	27 (71.0 %)	0.169
Completed (at least half)	12 (66.7 %)	23 (60.5 %)	0.658
Completed (all sessions available)^a^	3 (16.67 %)	8 (21.1 %)	0.268
Usual care attendance (*n* = 59)^b^			
DNA	13 (61.9 %)	15 (48.4 %)	0.337
Attended at least one session	8 (38.1 %)	16 (51.6 %)	0.336

*DNA* did not attend

^a^Fisher’s exact test was used to compare active vs passive recruitment instead of chi-squared test

^b^Seven missing values for usual care attendance information

### Qualitative data

Three-month interviews with parents/carers were carried out with 41 families allocated to Families for Health and 21 families allocated to usual care, including families who did and did not attend the intervention.

Families often spoke about how they came to join the study. The majority of families had already identified their child as being overweight and were either looking for support or had previously accessed services. The information about the study provided a further avenue of support at the right time for them:

*Yes, the City Show [where heard about the study]. I approached them [researcher] because I was quite concerned about her [daughter’s] weight and I saw they were measuring children … and talked about the problems I was having.* (Parent 33, Site C).

*Because at the time we were just battling with food so much that it was just getting too much for me. So I was looking for some help.* (Parent 20, Site B)

Some families spoke of how they had considered going to their GP for help with their child’s weight:

*I went into the doctor’s to ask for some help and I saw the leaflet and thought, rather than going through things and having on the medical records, I’d ask for some help and support that way [via study].* (Parent 101, Site B).

Some families had thought about their child’s weight but were not seeking help at the time they saw the advertisement about the trial:

*You [researcher] were in a tent there and … I thought, out of interest, my son is a bit on the big side… We didn’t know how he was getting on with centiles or anything. We just thought, visually to look at him, he is larger than what he should be compared to his peers… I went in there and got the measurements and they said, ‘He definitely is over what he should be.’ I found out about the programme there and then. ‘Are you interested?’ ‘Yeah, put us down, definitely. We’ll have a go.’* (Parent 39, Site B).

Receiving a letter about the study as part of the NCMP also acted as a catalyst for seeking help for some families:

*[Child A] had got weighed at school, part of the national weigh-in thing when they get into year 6. We got the results back and inside was a letter saying about her weight and there was this course or study going on with Warwick University, would you consider being part of it. I thought, well yes, I will do anything if it’s going to help her and all of us to lose the weight … even if we didn’t lose weight to give us ideas about changing lifestyle. Because I think sometimes the children just think oh it’s mum again, and I thought well maybe they might take more notice if it’s coming from somebody else and not just myself. So I thought yes, so I grasped it with two hands.* (Parent 112, Site A)

Several families spoke of how they were prompted to respond when they saw the information about the study for a second time:

*When I first saw in the school newsletter I thought, yes maybe, and then I didn’t do anything about it. And then I saw the second one and thought it was a sign so I ought to do something about it really. That was it.* (Parent 57, Site C).

## Discussion

We have found that passive methods of recruitment required fewer resources, generated more contacts and provided a higher percentage of contacts that converted into recruited families. However, without also using active methods of recruitment, the study would not have reached the recruitment target. For this study, it was important that we used both methods. Both methods had some success with families that were already looking for help about their child’s weight and those that were not.

At the time of study recruitment, there were more than 600 children in each of the three sites in year 6 alone (aged 10–11 years) who were eligible to take part based on their BMI from the NCMP 2008/9 [16]. We only required 40 from each locality, but nevertheless found it very difficult to recruit. In addition to being part of the Families for Health study, participants needed to be prepared to attend a time-consuming programme at a preset date and time. Our recruitment rates and our need to adjust timescales to reach the required participant numbers, demonstrate the challenges and complexity of recruitment [1]. The study required a 9-month extension (at no cost) to complete the 12-month follow-up of families.

Our finding that passive methods were more successful for recruitment to the trial is consistent with the studies by Nguyen [9] and Lee [17], albeit they differ from some previous research, where active recruitment methods yielded a higher number of study participants than passive [8]. An interesting finding in our study was that children of families recruited actively were more likely to be Asian or mixed race, possibly reflecting a higher prevalence of obesity in South-Asian children [18]. An alternative explanation is the increased association of body dissatisfaction and body fatness found in Asian children [19] so when identified that they were overweight they were more receptive to recruitment to the trial. A further difference in the baseline characteristics in the current study is that the parents’ mean BMI was higher in families recruited by passive methods, possibly indicating that parents’ own weight status also encouraged them to seek support for weight management.

The NCMP showed promise as a useful method of recruitment to the trial, and may account for why active methods resulted in a higher mean age of the children as NCMP is targeted at school year 6 (aged 10–11 years). However, this method can only be used when the timing is appropriate; that is, when measurement letters are being sent out at a time when recruitment to the intervention is ongoing. Future interventions should aim to synchronise their recruitment through NCMP.

Quantitative data on the number of times a family received information on the study was not collected, but the qualitative data suggest that families would often see a flyer more than once and it was a second viewing that prompted them to make contact. Passive recruitment can be more easily distributed on a larger scale and in multiple places, so is more likely to be seen by the intended audience. Our results suggest that repeat viewings of study information may be important. It is possible that active and passive recruitment methods could act synergistically to increase recruitment. Research in public health suggests that combining interpersonal communication with communication at an organisational or community level is more effective than mass media communication alone [20].

There was no significant association between the way in which a family was recruited and the likelihood of the family attending or completing the intervention (Table 2). While passive recruitment yielded a higher number of participants to the study, active recruitment also provided a substantial number of participants. Passive methods are usually cheaper and can target a larger number of people, but the reasons why a family may respond to a message about a child obesity treatment intervention are complex and may vary depending on ethnicity or parental BMI. This highlights the benefits of multiple recruitment strategies, including both those that aim at blanket coverage and those that are targeted at families with children who are overweight, to reach all the intended population. The implications for recruitment of families to future trials are:

- Passive recruitment can result in many enquiries and recruited participants. It is likely to be less costly per participant recruited.
- Families are receptive to information on childhood obesity trials posted in schools and GP practices.
- Media-led enquiries were low, but all families who enquired this way were recruited. Targeted media advertising may lead to good numbers of study participants.
- Seeing information about the study multiple times can encourage families to respond. Recruitment strategies need to aim for blanket as well as targeted coverage.
- Recruitment methods should be targeted to take into account the population, e.g. ethnicity.
- Use of NCMP for recruitment to child obesity trials shows promise. If the times of measurement of the child and recruitment to intervention are synchronised, recruitment may be more successful.
- Multiple strategies for recruitment to child obesity studies should be used.

## Conclusions

The systems by which a family is recruited to a group-based management intervention study for childhood obesity are complex and multiple recruitment strategies that are both passive and active, blanket and targeted, are likely to be necessary to achieve adequate sample sizes.

## Additional file

Additional file 1:
**Examples of recruitment methods.** (DOCX 2729 kb)

## Abbreviations

BMI: body mass index
DNA: did not attend
GP: general practice
HTA: Health Technology Assessment
NCMP: National Child Measurement Programme
NHS: National Health Service
NIHR: National Institute for Health Research
