# Supplementary material for: Evaluation of recruitment methods for a trial targeting childhood obesity: Families for Health randomised controlled trial
Source: Trials. 2015 Nov 25;16:535. doi: 10.1186/s13063-015-1062-x (PMC4660776; doi:10.1186/s13063-015-1062-x)

**Additional file 1: Examples of Recruitment Methods**

1. **ACTIVE METHODS**
2. **Letter from School Nurse Service following the NCMP (Site A)**

V1 30.4.12

Dear Parent/guardian

Your child recently had their height and weight measured as part of the National Child Measurement Programme

The results letter you received will have highlighted your child as being overweight or very overweight and informed you of services that would be able to offer you support if you were concerned.

We would like to make you aware of another programme that has become available in the city.

We enclose a leaflet with information about the service and how to contact them if you wish to find out more details about the programme.

Please note we have not shared any of yours or child’s details with the programme managers and are sending you this new information only to help you make an informed choice of services available if you want to take any further action.

The School Nursing Service can always offer one to one support and advice to any child and their family and please feel free to contact us if we can be of any help.

Regards

Team Leader

School Nursing Service

1. **Letter from Change4Life Service (Site B)**

Dear Parent/Guardian

Further to your child having their height and weight measured as part of the National Child Measurement Programme, you previously received a results letter and information about the Change4Life service available to you in [Site B]. We would also like to make you aware of another programme (Families for Health) that is now available in [Site B] and is currently part of a research study run by the University of Warwick.

We enclose a leaflet with information about the service and how to contact them if you wish to find out more details.

Please note, I work within the school health team and I have not shared any of yours or your child's personal details with the programme managers of this research trial.  I have addressed the envelopes myself using the information provided when the school health team weighed and measured your child.  I wanted to share the enclosed information with you in case you wanted an additional free of charge service for you and your child.  The programme manager will be able to give you more information about the trial once you have contacted them.

Regards

Family Change4Life Advi**s**or

School Health Team

1. **Letter from GPs to parents of eligible participants**

Version 2, dated 09.07.2013

Letter from GPs to potential participants (on GP headed paper)

GPs Name and Address

Dear

**`Families for Health' project for children who may be overweight**

This practice has agreed to support a research project being carried out by the University of Warwick to see if a family based programme can help children who are having problems with their weight. The programme is called *`Families for Health'* which is being compared with other support services available in the region. Families will be allocated, by chance, to receive either the *'Families for Health'* programme or the *'One Body One Life'* programme (if Site A GP) / 'Change 4 Life' advisors (if Site B GP) / support by the School Nurse or Weightwatchers (if child is 10 years) (if Site C GP) (delete as appropriate). Whichever programme families are allocated to, all families will receive free, local support.

According to our records, XXXX (name of child) may be overweight for his/her (delete as appropriate) height, and may benefit from this research project.

I enclose an information sheet about this research project. Please take your time to read this and to decide whether you are interested in taking part. If you would like more information and/or are interested in taking part in the research please complete the attached form, and send it directly to the researchers at the address provided at the bottom of the form in the stamped addressed envelope. Alternatively please contact the research team directly: Jo Kirby / Atiya Kamal on 024 76 151853 or email [FFHwarwick.ac.uk](http://FFHwarwick.ac.uk)

You do not have to take part. If you do not take part this will in no way affect the treatment you receive at the General Practice or from other healthcare teams.

Yours sincerely,

Dr

1. **PASSIVE METHODS**
2. **Poster**


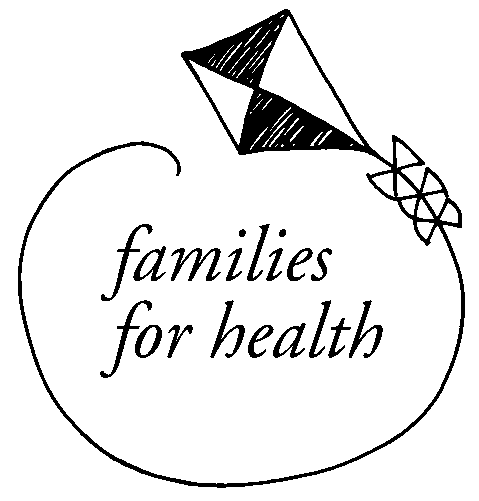


**FREE, LOCAL SUPPORT for families with an overweight child**

**We are looking for families to take part in a research study to test a new family-based programme for children who are overweight and aged between 6-11 years.**

**All families recruited to the programme will receive free, local support in a fun and family-friendly environment.**

**Your involvement could also benefit other families who are coping with this issue.**

**If you are interested in participating, or would like further information, please contact:**

**Email:** [**FFH@warwick.ac.uk**](mailto:FFH@warwick.ac.uk)

**Or call Jo Kirby/Atiya Kamal, on 024 76 151853, or**

**Wendy Robertson, on 024 76 574660**

|  | 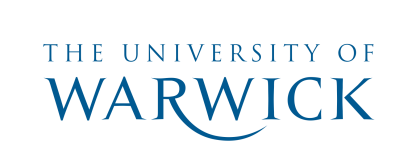 |  |
| --- | --- | --- |
|  |  |  |

1. **Text emailed to Schools for inclusion in their School Newsletter**

**Families for Health Programme**

Warwick University is currently looking for families to participate in a fun, new and local programme to support families with a child who is overweight. Children must be aged between 6 -11 years to be eligible. For information about this new family-based research please either email: FFH@warwick.ac.uk or call Jo Kirby on 02476151853.

1. **Example of Unpaid Media Coverage (in a Football Matchday Magazine)**


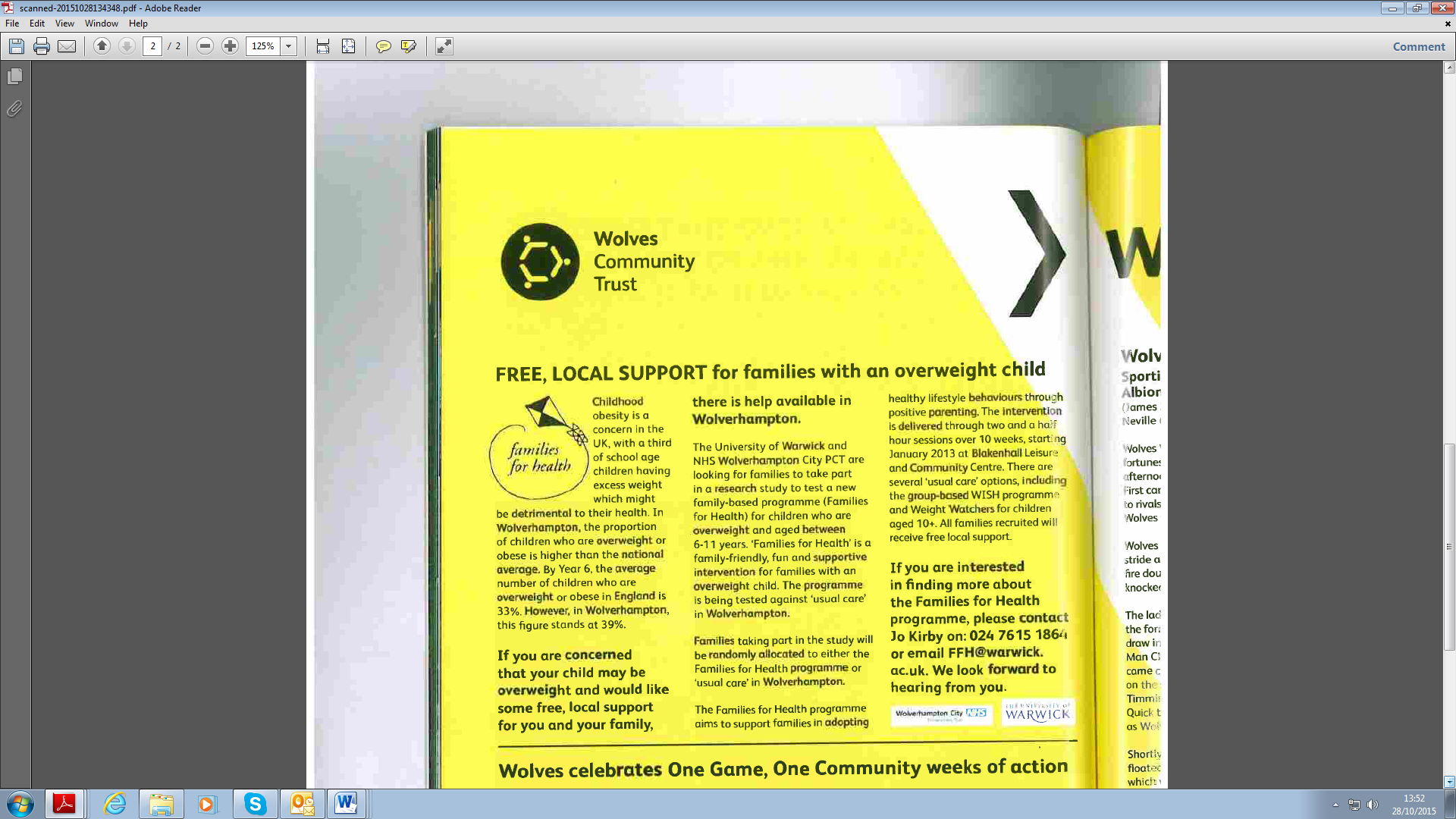


1. **Example of Unpaid Media Coverage (in a local newspaper)**

**
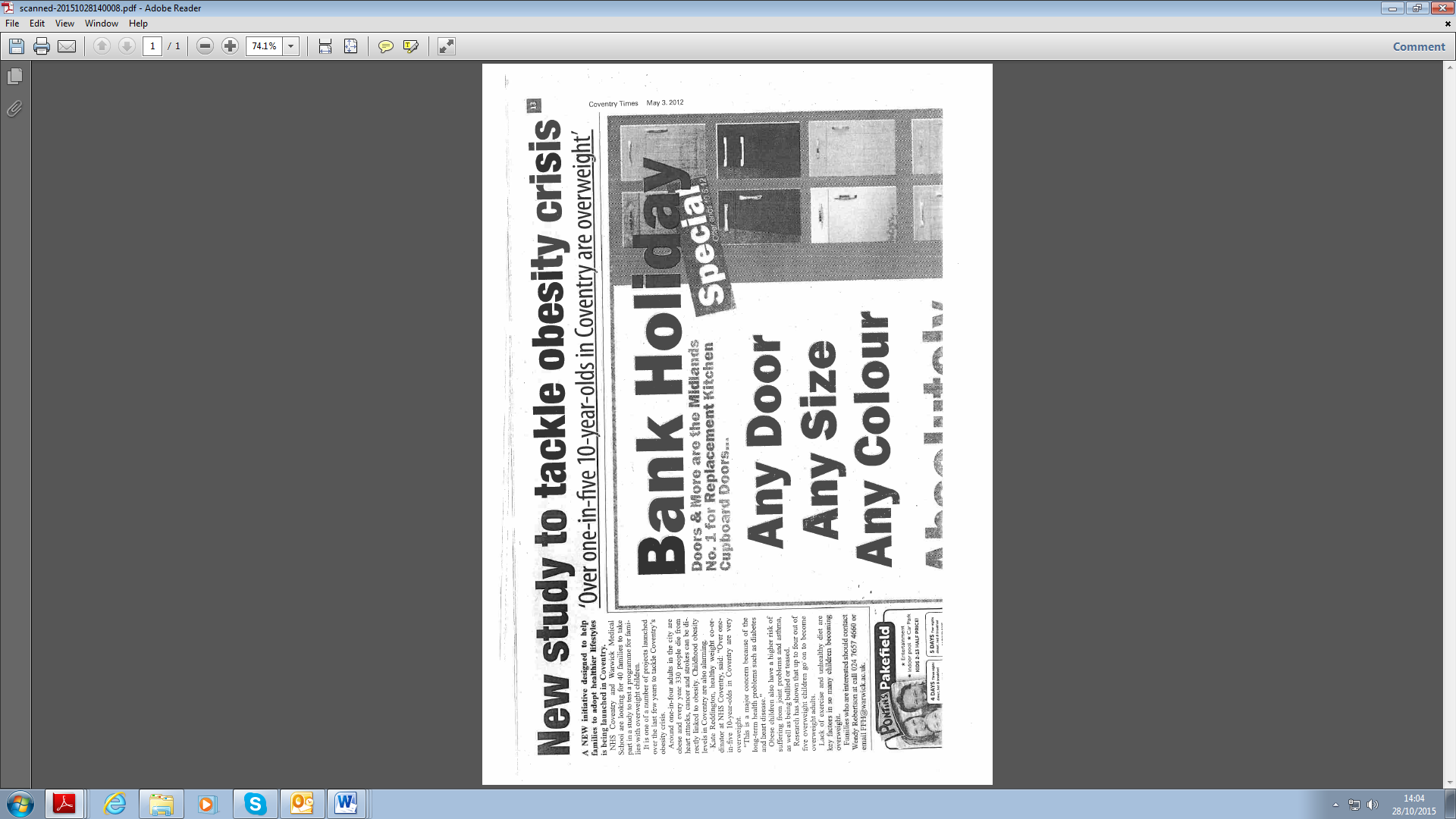
**

1. **Example of Unpaid Media Coverage (in a research newsletter)**
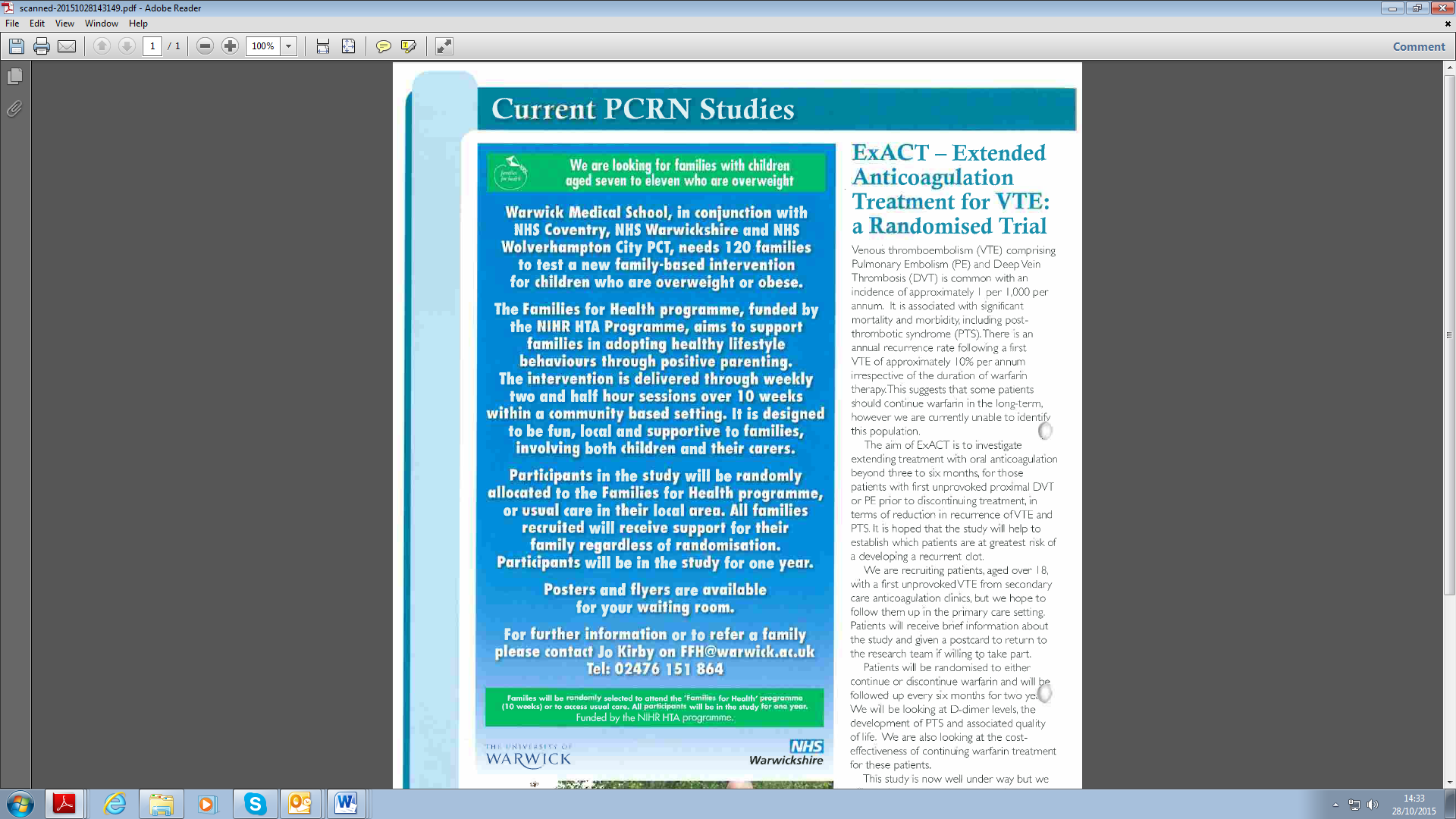

2. **Example of Paid Media Coverage**


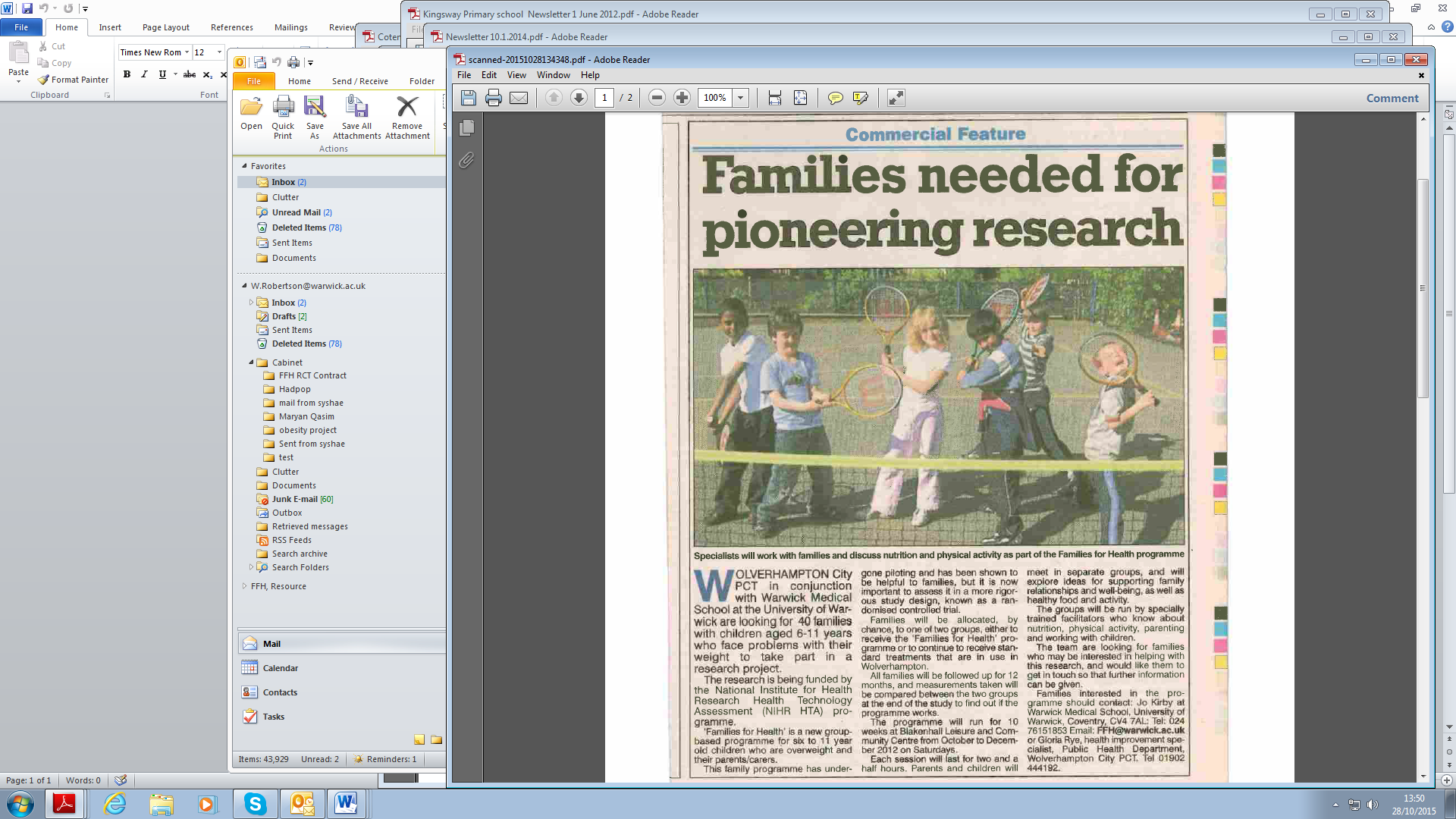

Supplement: Additional file 1: — Examples of recruitment methods. (DOCX 2729 kb) [file 13063_2015_1062_MOESM1_ESM.docx]
